# NIST-ASME WORKSHOP ON UNCERTAINTY IN DIMENSIONAL MEASUREMENTS Gaithersburg, MD June 5–7, 2001

**DOI:** 10.6028/jres.106.043

**Published:** 2001-10-01

**Authors:** Dennis A. Swyt

**Affiliations:** Precision Engineerings Division, Manufacturing Engineering Laboratory, National Institute of Standards and Technology, Gaithersburg, MD 20899-8210

## 1. Introduction

While the use of measurement uncertainty in dimensional measurements, particularly uncertainty represented according to the ISO *Guide to the Expression of Uncertainty in Measurement* (GUM), is a well-established practice among national metrology institutes (NMIs), is a required basis for commercial laboratory accreditation, and is being specified in international product standards, such use is rare in U.S. industry and problematic where used, being unsupported by documentary standards that could provide specific practical guidance. To help address the problem, Special Committee H213 of the Board of Standards of the American Society of Mechanical Engineers (ASME) and the Manfacturing Engineering Laboratory of the National Institute of Standards and Technology (NIST) co-sponsored the workshop which this report describes. The conference organizers were: Dr. Gregory Hetland, Hutchinson Technology Incorporated; Dr. Jay Raja, University of North Carolina, Charlotte; and Dr. Dennis Swyt, NIST, who acted as conference moderator.

## 2. Purpose of Workshop

The purpose of the 2 1/2 day workshop was two-fold. The first purpose was to inform participants on trends and problems associated with the use of measurement uncertainty in industrial applications from a variety of perspectives, particularly problems addressable by documentary standards-based recommended practices. The second purpose was for participants to develop recommendations, particularly to the documentary standards community, on potential solutions to identified problems.

## 3. Participants of the Workshop

The intended audience for the workshop was industrial and laboratory metrologists, engineers, and managers with interests in the use of statements of uncertainty for results of dimensional measurements for any purpose. Notification of potential attendees was via NIST’s conference announcement process, the NCSL-I^1^ newsletter, and a broad mailing to membership lists of ASME standards committees, the American Society for Precision Engineering, and NIST dimensional calibration customers. Fifty-six persons from the following organizations participated in the workshop:

A2LA
Adcole
AH Inc
Axeon
Boeing
Bosch Brakes
Caterpillar
Daimler-Chrysler
Dyncorp
Fairchild
Gagedoctor
Hutchinson Technology
Mahr Federal
Mitutoyo
NIST
NRC-Canada
Pratt & Whitney
S Cal Edison
UNC Charlotte
US Air Force
US Army
Worcester Polytech
Wyle Laboratories

## 4. Structure of Workshop

The workshop was structured to provide participants with reports on practices and problems in the use of uncertainty in dimensional measurements and an opportunity to make recommendations on potential solutions to the problems identified. The first day was devoted to presentations on practices and problems in the use of uncertainty in dimensional measurements from different points of view. The following list shows the topic, speaker, and affiliation for each of the 12 presentations:

**Table:** 

U.S. national documentary	J. Salsbury	Hutchinson Technology
Measuring-instrument supplier	J. Salsbury	Mitutoyo
Aircraft manufacture	B. Parry	Boeing
Auto manufacture	J. Fallert	Daimler-Chrysler
Computer equipmentmanufacture	J. Buttress	Hutchinson Technology
Microelectronics manufacture	R. Scace	NIST/OMP
Industry standards laboratories	K. Jaeger	NCSL-I
International documentary standards	T. Charlton	ASME H213
U.S. national documentary standards	T. Estler	NIST/ASME B89.7
National Measurement Institute	J. Decker	NRC-Canada
R&D on coordinate metrology	S. Phillips	NIST
Laboratory accreditation	S. Doty	NVLAP
VIM-GUM	T. Estler	NIST/JCGM

The second day of the workshop was devoted successively to breakout sessions for identification of problems in the use of uncertainty in dimensional measurements, a plenary session for review of problems identified, and breakout sessions for development of proposed solutions to problems. Breakout sessions were facilitated by T. Estler, J. Kramar, S. Phillips, J. Stoup, and D. Swyt of NIST. The third (half) day was devoted to plenary-session development of rank-ordered recommendations on problems identified, including follow-up actions for individual workshop participants.

## 5. Results of Workshop

The following are the seven resulting rank-ordered statements of identified problems, proposed solutions, and recommended actions to be coordinated by ad hoc workshop follow-up committee lead by workshop organizers.

### 5.1 Steering Group to Lead in Measurement Uncertainty Education

#### Problem

While (1) international standards bodies are developing specifications requiring use of measurement uncertainty, (2) domestic manufacturers anticipate growing need for their use and (3) calibration laboratories seeking accreditation must use them to establish required traceability, manufacturers point to the lack of a body of people who are trained to understand and knowing how to use uncertainty as these specifications require. Laboratories, on the whole, are afraid of the term “uncertainty budget” because they do not know how to do calculate one, and educators note a lack of easily accessible resource materials to educate properly current and future practitioners in measurement uncertainty. Further, while virtually everyone, from NMI to industrial practitioner, needs to produce statements of uncertainty, many do not understand the instruments and measurement processes well enough and do not have the resources to acquire the understanding needed to do so. In addition, no single entity has overall responsibility for the problems of “training and education” in the area of uncertainty in dimensional measurements.

#### Solution

Under the leadership of an ad-hoc steering group:

- Establishment of a group (possibly composed of high-level managers from industry, government agencies, and universities) with the mission to promote the long-term sustainable educationa efforts in the use of uncertainty in measurements, particularly dimensional measurements.
- Establishment of a group (possibly within ASME B89.7, NCSLI, NIST, or universities) with the mission to catalogue currently available educational resources for uncertainty issues.
- Development of an “educational framework,” that is, a comprehensive vision and scheme for education in measurement uncertainty, covering
  what needs to be known (general: statistics, cost/benefit ratios, VIM/GUM, metrology, geometric dimensioning and tolerancing, mathematics, physics; industry-specific: heavy manufacturing, light manufacturing, precision manufacturing, semiconductor, electrical)
  who needs to know it (practitioners, assessors, financial managers, technical managers, NMIs, researchers, customers)
  who will fund it (NSF, NIST, DOE, DOD, CCG, SME, ASQ, industrial consortia, students, industry lobby groups)
  how it can be provided (self-paced learning, commercial training classes, websites, free modules for university training)
  who will provide it (vendors, consortium of universities and national labs, professional societies, companies)
  when it should be provided (high school instructions through bridge programs/internships; post-secondary education; vocational/trade schools, junior colleges, universities, just-in-time training, continuing education)
  how it will be validated/certified (multi-level certification by professional societies, e.g., ASME, SME, NCSLI, SAE, AMTMA, ASQ, vendors, colleges, universities).

### 5.2 Consortium Devoted to Meeting Industrial Needs in Measurement Uncertainty

#### Problem

The cost-burden on individual, especially small, companies is too great (at this time) for them to support the development of education, training, communication, and standards-development infrastructure needed to effectively and promptly address the wide range of such problems in the use of uncertainty in measurements, particularly dimensional measurements, facing U.S. industry today.

#### Proposed Solution

Establishment of an industrial consortium dealing with problems in the use of uncer tainty in measurements, particularly dimensional measurements, in order to leverage dollar investments by individual members of the consortium and to pursue other sources of funding, including from Congress, the NIST ATP Program, NSF and the like.

### 5.3 Website for Measurement Uncertainty Documents

#### Problem

While there are many documents on uncertainty in dimensional measurements, (including international and national standards, accreditation practices, and technical reports) of potential value to a broad range of possible users, these documents are scattered and not easily accessible to many, with no easy and efficient means by which one may find out about them.

#### Proposed Solution

Creation and operation by an appropriate institution of an “Uncertainty in Dimensional Measurements” web site focussed on the principal documents on the subject. Proposed features of the website include: optional registration; a hierarchy of documents; diagram of standards, and guides showing interrelationships; synopses of documents; user reviews of documents; news of upcoming round-robins; user forum; case studies; testimonials to cost savings and continuous improvement; a roadmap on developments; and a primer for neophytes.

### 5.4 Business-Case Justifications on Use of Measurement Uncertainty

#### Problem

The business community requires cost justification for allocating dollars to implement measurement uncertainty. For example, executives and managers need a basis for spending on training and devoting human resources to support analysis and use of measurement uncertainty and purchasing agents need a basis to weigh the uncertainty versus cost in acquiring measuring instruments.

#### Solution

Development by ASME B89.7 of technical reports that provide business-case justification for the use of uncertainty in measurements, including

- “problem statement paper covering ROI opportunities” dealing with
  decision rules (false rejects, false accepts)
  calibration frequency extended life of measurement systems
  product warranty risks quantified
  improved process to achieve competitive advantage
  allocation of limited resources for highest ROI (CMM, lab, fixture, sampling, cleaning)
  recovery of yields loss
- ROI case studies as published technical reports
  ROI Case Study 1 on selecting measuring equipment (including system, room, cleaning, allocating resources; impact of required infrastructure from industrial labs to NIST; determining calibration frequency and control parameters; reducing buyer/seller disagreement)
  ROI Case Study 2 on dealing with measurement uncertainty with respect to tolerances at the design stage (including recovering yield loss through decision rules, process control and impacts of measurement, e.g., Motorola Six-Sigma Quality)
  ROI Case Study 3 on Failure Mode and Effects Analysis (Catastrophic failures, e.g., Shuttle Challenger, Ford-Firestone, Dodge Minivan latches, seatbelt recalls and lawsuits, scrap of high-cost products).

### 5.5 Accreditation-Related Standard Methods on Uncertainty

#### Problem

In the area of accreditation (which is defined broadly to include accreditation, certification, and auditing), there is an absence of documented standard methods related to measurement uncertainty for use by both auditors of laboratories and laboratories being audited. While many institutions—as part of a system—create, invoke, or require procedures on the use of measurement uncertainty and end-users of such procedures may have to deal with inconsistent requirements, no single institution has responsibility in the system for producing a single, self-consistent, technically-sound set of standard procedures that may be used by all.

#### Proposed Solution

The proposed solution is the coordinated development by ASME, NCSLI, NIST, NACLA (and other institutions with shared responsibility in the area of measurement uncertainty) of documented standard methods for: itemizing and listing contributions to uncertainty that must be addressed; appropriately defining “scope of accreditation,” e.g.. for coordinate metrology; evaluating the credibility of uncertainty statements; proficiency testing of on-site (non-laboratory) calibrations; formatting uncertainty budgets; and demonstrating traceability in practice.

### 5.6 Addressing of Specific Technical Issues by Standards Committee

#### Problem

There is an array of problems associated with current and needed documentary standards governing the use of uncertainty in dimensional measurements, including:

The GUM, its complexity for most people to use, and its unsuitability for economic decision-making
The establishment of credibility and validity of uncertainty statements
Identification of significant input quantities
Inconsistency in reporting format
Inconsistency in terminology/vocabulary and interpretation
Decision rules connected to economics
Calibrated instruments used for unlike measur ands and/or extended conditions
Evaluation of measurement uncertainty for calibrations based on a single observation
Measurands that are insufficiently defined by some standards (e.g., ISO 1101)
Lack of consensus standards on some instrument performance specifications/tests.

#### Proposed Solution

- Development by the international committee responsible for the GUM (JCGM/WG1/SC4) of models for measurement uncertainty that are simple, general, and usable by industry
- Development by the U.S. national standards committee responsible for uncertainty in dimensional measurements (ASME B89.7) of documentary standards, including formal standards or technical reports, that
  provide a simplification of the GUM more usable by industry than the GUM itself (B89.7.3.2)
  define a basis for establishing the credibility and validation of statements of uncertainty in dimensional measurements (B89.7.3.3)
  identify the significant input quantities to uncertainty in dimensional measurements (B89.7.3.2)
  standardize the reporting format for statements of uncertainty, uncertainty budgets, expected sources of uncertainty, and the criteria for consideration of those expected sources within the reporting format (B89.7.3.1)
  provide decision rules connected to economics for the use of measurement uncertainty in, for example, determining conformity of manufactured parts or measuring instruments to specifications (B89.7.3.1)
  deal with instruments that are calibrated for one measurand under one set of measurement conditions being used for other measurands and/or other conditions [see “Careful Consideration of the Calibration Concept,” M. S. Levenson et al., J. Res. Natl. Inst. Stand. Technol. **106**, 371 (2001)] deal with evaluating measurement uncertainty for calibrations or measurements based on a single observation (measurement), rather than repeated ones, by GUM Type B assessment (B89.7.4)
- Action by institutions with shared responsibility in use of measurement uncertainty (ASME, ASQ, NCSLI, NIST, NRC-Canada, …) to
  promote use of International Vocabulary of Metrology (VIM)
  identify inconsistencies in terminology, vocabulary, and interpretation within their respective domains
  identify dimensional-metrology instruments lacking consensus
  standards on uncertainty-related performance specifications and testing
  inform appropriate standards committees of such inconsistencies in terminology and absence of needed instrument performance tests.

### 5.7 Establishment of Sound Practice on Technical Qualification of Assessors

#### Problem

Variability in technical qualifications of laboratory accreditation assessors and/or technical experts resulting in inconsistencies among accredited laboratories compromises (in some cases) the actual or perceived integrity of the laboratory accreditation process.

#### Solution

Development and application by NACLA (National Cooperation for Laboratory Accreditation) of sound practice and a uniform method for the specification and verification of the technical qualifications of assessors, whether they be the assessors themselves or the technical experts that operate with formal assessors in the laboratory accreditation process.

### 5.8 Operational Follow-Up to Recommendations

As an operational follow-up recommendation, the participants in the workshop together recommend the establishment by the organizers of the workshop of an “Ad-Hoc Group on Measurement Uncertainty in Dimensional Measurements” made up of the organizers, volunteers from among the other participants of the workshop, and other interested parties to pursue by appropriate actions the seven other recommendations of the workshop.

The participants of the workshop also suggest:

a prompt follow-up meeting of workshop participants and others
posting of workshop presentations immediately on the NIST/PED website
a publicity article on the workshop by the organizers in appropriate journals such as a Conference Report in the Journal of Research of NIST.

The following workshop participants volunteered or were nominated to take follow-up actions on the various recommendations of the workshop:

1. establishment of steering group on education in measurement uncertainty, including proposal to NCSL to take special role in steering group Steve Stahley, Walt Lehmus, Jim Ferguson, Ed Morse
2. establishment of a consortium on meeting industrial needs Rob McNaughton (to work, not lead)
3. establishment of a website on documentary information Jay Raja (nominated)
4. development of business-case justifications on use of uncertainty John Buttress representing ASME B89.7 committee
5. development of accreditation-related standard methods (workshop organizers by default)
6. addressing specific technical issues by a standards committee (workshop organizers by default)
7. establishment of sound practice on qualification of assessors (workshop organizers by default).

## Footnotes

1 See Appendix A for explanations of acronyms.

## 6. Appendix A. Acronyms

**Table:**

AMTMA	American Measuring Tool Manufacturers Association
ASME	American Society of Mechanical Engineers
ASQ	American Society for Quality
BIPM	Bureau Internationale des Poids et Mesures
CCG	Calibration Coordinating Group
CMM	Coordinate Measuring Machine
DOD	Department of Defense
DOE	Department of Energy
FMEA	Failure Mode and Effects Analysis
GUM	ISO *Guide to the Expression of Uncertainty in Measurement*
HTI	Hutchinson Technology Incorporated
ISO	International Organization for Standardization
JCGM	BIPM Joint Committee for Guides in Metrology
NACLA	National Cooperation for Laboratory Accreditation
NVLAP	National Voluntary Laboratory Accreditation Program
NCSL-I	National Conference of Standards Laboratories International
NIST	National Institute of Standards and Technology
NMI	National Metrology Institute
NRC	National Research Council of Canada
NSF	National Science Foundation
OMP	NIST Office of Microelectronic Programs
PED	NIST Precision Engineering Division
ROI	Return on Investment
SAE	Society of Automotive Engineers
SME	Society of Manufacturing Engineers
UNC-C	University of North Carolina—Charlotte
VIM	International Vocabulary of Basic and General Terms in Metrology.

